# Laparoscopic Heller Myotomy and Dor Fundoplication for the Treatment of Esophageal Achalasia After Sleeve Gastrectomy—a Video Vignette

**DOI:** 10.1007/s11695-020-05114-x

**Published:** 2020-11-24

**Authors:** Alberto Aiolfi, Diego Foschi, Marco Antonio Zappa, Alessandra Dell’Era, Emilia Bareggi, Emanuele Rausa, Giancarlo Micheletto, Davide Bona

**Affiliations:** 1 Department of Biomedical Science for Health, Division of General Surgery Istitituto Clinico Sant’Ambrogio, University of Milan, Via Luigi Giuseppe Faravelli, 16, 20149 Milan, Italy; 2 Department of Biomedical and Clinical Sciences “Luigi Sacco”, L. Sacco Hospital, Università degli Studi of Milan, 20157 Milan, Italy; 3 ASST Fatebenefratelli Sacco, UOC di Chirurgia Generale, Milan, Italy; 4 General Surgery I, Papa Giovanni XXIII Hospital, Bergamo, Italy; 5 Department of Pathophysiology and Transplantation, INCO and Department of General Surgery, Istituto Clinico Sant’Ambrogio, University of Milan, Milan, Italy

**Keywords:** Laparoscopic sleeve Gastrectomy, Heller myotomy, Dor fundoplication, Video vignette

## Abstract

**Purpose:**

Esophageal dysmotility and disorders of the lower esophageal sphincter are well documented in morbidly obese patients. Esophageal achalasia has been reported in up to 1% of obese patients but the development of such esophageal motility disorder after laparoscopic sleeve gastrectomy (LSG) is extremely rare. The purpose of this video was to demonstrate the management of a type II esophageal achalasia diagnosed in a 46-year-old female patient 4-year after LSG.

**Materials and Methods:**

An intraoperative video has been anonymized and edited to demonstrate the feasibility of laparoscopic Heller myotomy and anterior Dor fundoplication on the mentioned patient.

**Results:**

The operation started with the section of the perigastric adhesions. Proceeding in a clockwise direction, the esophagogastric junction, the anterior esophageal wall, and the His angle were freed. A residual slightly dilated fundus was found and isolated. After mobilization of the distal esophagus and identification of the anterior vagus nerve, a “hockey stick” myotomy was carried out for 6 cm on the esophagus and for 2 cm on the gastric side. An anterior Dor fundoplication was fashioned using the residual gastric fundus.

**Conclusion:**

Esophageal achalasia in patients that previously underwent LSG is exceptional but should always be suspected in case of pathognomonic symptoms onset. In tertiary referral centers, laparoscopic Heller myotomy and, if technically feasible, an anterior Dor fundoplication seem safe and effective to relieve gastroesophageal outflow obstruction and prevent gastroesophageal reflux.

**Supplementary Information:**

The online version contains supplementary material available at 10.1007/s11695-020-05114-x.

## Introduction

Achalasia has been reported in up to 1% of morbidly obese patients [1]. The onset and diagnosis of esophageal achalasia arising after bariatric surgery is extremely rare [2, 3]. While the onset of this motility disorder after Roux-en-Y Gastric Bypass (RYGB) seems more common, only four cases of esophageal achalasia after LSG have been reported in the literature [4]. The management of such patients is challenging; however, it is likely that because the increasing number of morbidly obese patients and concomitant increase in weight-loss procedures (especially LSG), the number of these patients will growth in the future.

## Purpose

The purpose of this video was to demonstrate the management of esophageal achalasia in a 46-year-old female patient that underwent LSG for morbid obesity (weight 137 kg; BMI 39.8 kg/m2). The patient was referred to our institution 4 years after the index procedure (weight 46 kg; BMI 20.1 kg/m^2^) for worsening solid food and paradoxical dysphagia (Eckardt score 2-2-1-2) with anomalous ongoing weight loss. The high resolution manometry was suggestive for a type II achalasia with esophageal panpressurization.

## Methods

An intraoperative video has been edited to demonstrate the feasibility of a patient-tailored laparoscopic Heller myotomy and Dor fundoplication. Written informed consent was obtained from the patient.

## Results

**Fig. 1:**
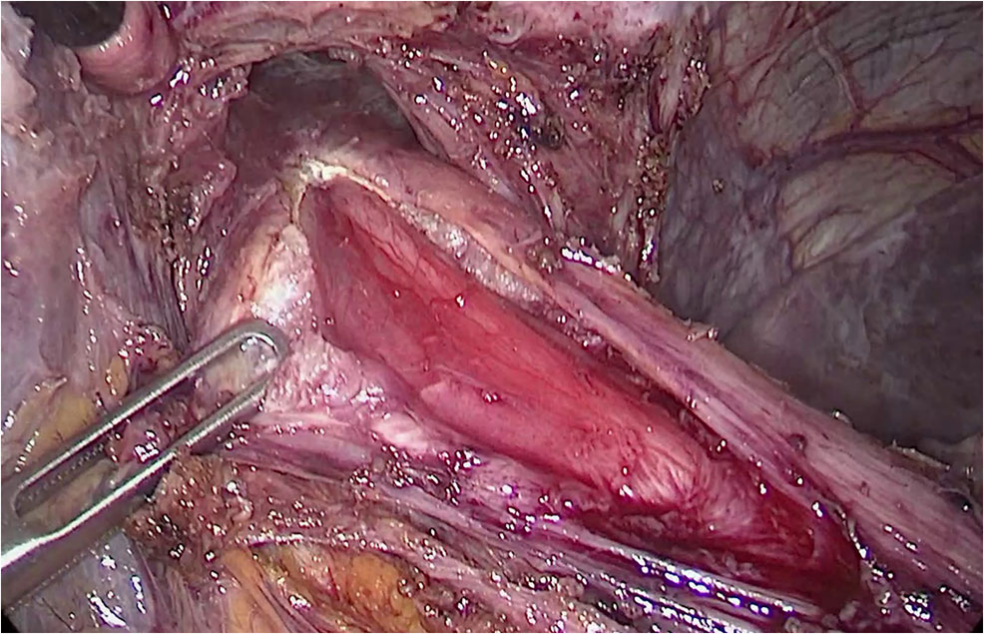
The myotomy is performed by sectioning the circular and longitudinal muscular layers 6cm on the esophagus. The myotomy is then extended 2cm distally below the esophagogastric junction. The underlying mucosa is exposed

The operation started with the section of the perigastric adhesions. Proceeding in a clockwise direction, the esophagogastric junction, the anterior esophageal wall, and the His angle were freed. A residual slightly dilated fundus was noticed and freed from posterior adhesions. The distal esophagus was mobilized and the anterior vagus nerve identified. A “hockey stick” myotomy was carried out for 6 cm on the esophagus and for 2 cm on the gastric side to include the oblique fibers (Helvetius collar) (Fig. 1). Intraoperative endoscopy with careful insufflation was negative for air leak. An anterior Dor fundoplication was fashioned by using the residual fundus that was secured to the left and right edge of the myotomy with interrupted non-absorbable sutures (Prolene ®—Ethicon, Johnson & Johnson, NJ, USA). The operative time was 85 min. The postoperative course was uneventful and the patient was discharged on postoperative day 2. At 12-month follow-up, the patient was asymptomatic on a single-dose proton pump inhibitor and the 24-h pH-impedance test showed no evidence of pathologic reflux.

## Discussion

The issue of whether an antireflux procedure should be added to the Heller myotomy has long been controversial. A 2004 randomized clinical trial showed the superiority of the myotomy plus Dor fundoplication versus myotomy alone with a significantly reduced postoperative pathologic reflux and esophageal acid exposure [5]. In accordance with these findings, fundoplication should be recommended after myotomy. None of the preoperative exams described the presence of a slightly dilated fundus and the decision to perform a Dor fundoplication was intraoperative and patient-tailored. The residual fundus allowed the fashioning of an anterior tension-free fundoplication with respect to valve geometry and lack of esophageal twisting. Furthermore, because of rapid and ongoing achalasia-related weight loss, the conversion to a RYGB to prevent gastroesophageal reflux would have further worsened this condition.

The reported rate of mucosal perforation during Heller myotomy is up to 5% and dependent on surgeon’s expertise [6]. We recommend endoscopic evaluation or air leak test via nasogastric tube as appropriate to check the integrity of the myotomy during the learning curve. In our referral center, endoscopy is used at surgeon discretion in challenging cases or after stitch repair for mucosal perforation.

## Conclusion

Esophageal achalasia after LSG is exceptional but should always be suspected in case of pathognomonic symptoms onset. In tertiary referral centers, a patient-tailored laparoscopic Heller myotomy and, if technically feasible, Dor fundoplication seem safe and effective to relieve symptoms and prevent gastroesophageal reflux.

## Supplementary Information

ESM 1 (MP4 352,002 kb).
